# Human endogenous retrovirus W in multiple sclerosis: transcriptional activity is associated with decline in oligodendrocyte proportions in the white matter of the brain

**DOI:** 10.1007/s13365-024-01208-9

**Published:** 2024-05-08

**Authors:** Tapio Nevalainen, Arttu Autio-Kimura, Mikko Hurme

**Affiliations:** 1 Faculty of Medicine and Health Technology, Tampere University, Arvo Ylpön katu 34, 33520 Tampere, Finland; 2 Gerontology Research Center (GEREC), Tampere, Finland; 3 Tampere University Hospital, Tampere, Finland

**Keywords:** Human endogenous retroviruses, HERV-W, Multiple sclerosis, RNA-sequencing, CIBERSORTx

## Abstract

Multiple sclerosis (MS) is a chronic inflammatory demyelinating disease. One of the basic mechanisms in this disease is the autoimmune response against the myelin sheet leading to axonal damage. There is strong evidence showing that this response is regulated by both genetic and environmental factors. In addition, the role of viruses has been extensively studied, especially in the case of human endogenous retroviruses (HERVs). However, although several associations with MS susceptibility, especially in the case of HERV-W family have been observed, the pathogenic mechanisms have remained enigmatic. To clarify these HERV-mediated mechanisms as well as the responsible HERV-W loci, we utilized RNA sequencing data obtained from the white matter of the brain of individuals with and without MS. CIBERSORTx tool was applied to estimate the proportions of neuronal, glial, and endothelial cells in the brain. In addition, the transcriptional activity of 215 HERV-W loci were analyzed. The results indicated that 65 HERV-W loci had detectable expression, of which 14 were differentially expressed between MS and control samples. Of these, 12 HERV-W loci were upregulated in MS. Expression levels of the 8 upregulated HERV-W loci had significant negative correlation with estimated oligodendrocyte proportions, suggesting that they are associated with the dynamics of oligodendrocyte generation and/or maintenance. Furthermore, Gene Set Enrichment Analysis (GSEA) results indicated that expression levels of three upregulated HERV-W loci: 2p16.2, 2q13, and Xq13.3, are associated with suppression of oligodendrocyte development and myelination. Taken together, these data suggest new HERV-W loci candidates that might take part in MS pathogenesis.

**Supplementary Information:**

The online version contains supplementary material available at 10.1007/s13365-024-01208-9.

## Introduction

The myelin sheath plays a crucial role in facilitating fast axonal signal transmission and preventing signal loss or interference, thus enabling efficient neural communication (Frankenhaeuser and Schneider 1951). Moreover, myelin sheaths actively contribute to supporting axonal energy needs by producing adenosine triphosphate (ATP) (Ravera and Panfoli 2015). Oligodendrocytes, the cells responsible for producing the myelin sheaths in the central nervous system (CNS), play a crucial role in the continuous maintenance of myelin integrity (Aber et al. 2022) but they also actively support axonal function and survival (Nave and Trapp 2008).

Multiple sclerosis (MS) is an autoimmune disease characterized by inflammatory processes that gradually degrade myelin sheaths surrounding CNS axons (Wu and Alvarez 2011). Consequently, the loss of myelin sheath exposes axons, rendering them vulnerable to degeneration (Simkins et al. 2021). While white matter is predominantly affected by demyelination (Trapp et al. 2018), spinal cord involvement is also observed (Marrodan et al. 2020). These demyelinating events occur within focal lesions with variable regional distribution, contributing to the diverse spectrum of symptoms (Ghasemi et al. 2017).

The precise etiology and underlying mechanisms driving the development of MS remain incompletely elucidated. However, a well-established phenomenon in the pathogenesis of MS is the infiltration of peripheral lymphocytes across the blood–brain barrier (BBB) into the CNS. This infiltration sets off a cascade of inflammatory responses within the CNS, leading to demyelination and axonal degeneration (Rodríguez Murúa et al. 2022). Autoreactive lymphocytes targeting CNS autoantigens are thought to be involved in this process (Liu et al. 2022). It has also been proposed that demyelination in MS may involve multiple mechanisms. These mechanisms include T cell–mediated demyelination, antibody–mediated demyelination and oligodendrocyte death (Cudrici et al. 2006; Staugaitis and Trapp 2012). Additionally, CNS-resident cells, such as astrocytes and microglia, play significant roles in MS pathogenesis (Absinta et al. 2021).

The onset of MS is thought to be determined by the interplay between genetic predisposition and environmental factors (Waubant et al. 2019). Among the various environmental triggers, viral infections, such as Epstein-Barr virus (EBV), human herpesvirus 6 (HHV-6), and varicella-zoster virus (VZV) have been associated with the onset of MS due to their potential mechanism involving molecular mimicry between viral antigens and CNS autoantigens (Libbey et al. 2007). Human endogenous retroviruses (HERVs), remnants of ancient retroviral infections integrated to human genome millions of years ago, have also been linked to MS development and progression. The human genome harbors an estimated 100,000 individual HERV fragments that constitute about 8% of our genetic material (Belshaw et al. 2004). The prototypic HERV sequence consists of four main retroviral genes (*gag, pro, pol, env*) that code for structural proteins, viral enzymes, and envelope glycoproteins (Grandi and Tramontano 2018a). It is worth noting that HERV open reading frames (ORFs), that contain the viral genes, have undergone extensive mutations and deletions over time, rendering them incapable of producing viral particles (Grandi and Tramontano 2018b). However, numerous HERV elements are transcriptionally active, producing RNA, across tissues (She et al. 2022). A small subset of these elements can produce full-length or truncated proteins that may have physiological functions or contribute to disease mechanisms (Grandi and Tramontano 2018a).

Especially the HERV-W subfamily of HERVs has been of particular interest in the context of MS. Transcriptional activity of numerous HERV-W loci in the brain has been reported in both normal conditions and disease states (Schmitt et al. 2013; Elkjaer et al. 2021). Research has also indicated an increased expression of the HERV-W envelope protein (env) within lesions found in individuals with MS (Grandi and Tramontano 2018a). This protein is believed to originate from one or a few specific well-preserved HERV-W loci and it has been shown to have pro-inflammatory effects, contributing to the disruption of the blood–brain barrier and myelin destruction (Kremer et al. 2019). There is also evidence that HERV-W env protein is cytotoxic to oligodendrocytes through the induction of the release of redox reactants (Antony et al. 2004). While 7q21.2 (syncytin-1) and Xq22.3 have been identified as primary candidate loci due to their structural integrity, there is still uncertainty about the origin of the env protein. Human genome harbors at least 13 HERV-W loci that have full-length env, but it has been speculated that HERV-W loci with truncated and mutated env genes might produce shorter proteins that could also have a role in disease (Grandi et al. 2016). While the pathogenicity of the HERV-W in MS is commonly attributed to the env protein, research has also shown that HERV-W RNA can induce neuroinflammation by activating NF-κB and triggering the production of type 1 interferons (Morris et al. 2019; Rangel et al. 2022; Russ and Iordanskiy 2023). Consequently, in the context of MS, a broader investigation including HERV-W loci without protein-coding potential may hold significance.

In this study, we utilized RNA sequencing data to analyze the transcriptional activity of 215 distinct HERV-W loci in the white matter of the brain comparing MS and control samples. In addition, we employed a transcriptional deconvolution technique to estimate the proportions of neuronal, glial, and endothelial cells and analyzed whether there are associations between transcriptional activity of HERV-W loci and cell proportions. This approach could provide valuable insights into pinpointing the HERV-W loci associated with MS pathogenesis, while also shedding light on associated mechanisms.

## Materials and methods

### Samples

Sample population comprised RNA sequencing data of white matter brain samples sourced from the Gene Expression Omnibus (GEO) database (GSE138614). The dataset consisted of a total of 98 samples that originated from 10 individuals with MS and 5 control patients. The biological samples had been obtained postmortem, and the mean age of the patients and controls were 52.4 and 56.4 years, respectively. Range of disease duration was from 10 to 50 years (mean 26.7 years). Time in progressive phase varied between 2 and 21 years (mean 13.75 years). Men and women were equally represented. More specific sample description can be found in (Elkjaer et al. 2019). Only 72 samples were used in this study. 26 samples were filtered out due to low sequencing depth, which did not enable reliable HERV quantitation. Of the 72 samples, 52 were from the patients with MS, and 20 samples were extracted from controls without neurological disease. Furthermore, the MS samples were obtained from the patients with varying stages of the MS. The classification was either “active”, “inactive”, “chronic active”, “remyelinating”, or “normal appearing white matter”. The number of samples from each class was 11, 10, 11, 3, and 17, respectively. This classification information, however, was not used to avoid sample sizes becoming too small.

### Cellular deconvolution analysis

The proportions of neuronal, glial and endothelial cells were estimated using deconvolution analysis with the tool CIBERSORTx (Newman et al. 2019). CIBERSORTx utilizes support vector regression and gene expression data to estimate relative abundances of predefined cell types from the mixed cell type sample. CIBERSORTx has been validated with immunohistochemistry (Saito et al. 2022) and simulated datasets (Sutton et al. 2022). The cell types of interest and their marker genes are defined in a signature matrix file, which has to be well defined. In this study we utilized signature matrix provided by Wang et al. (2020), which defines gene expression signatures for astrocytes, oligodendrocytes, microglia, excitatory and inhibitory neurons, and endothelial cells. Prior to deconvolution, the RNA-seq counts were CPM normalized and log2 transformed to match the data processing of the signature matrix. CIBERSORTx was run with batch correction enabled, which is applied prior to deconvolution analysis. For significance analysis, the number of permutations was set to 1000.

### HERV-W RNA expression quantification

The HERV-W annotations used in this study originate from a curated list provided by Grandi et al. (2016). Computational analyses were run in Puhti supercomputer of CSC (Espoo, Finland). SRA Toolkit (v2.10.8) was used to transfer unprocessed RNA-sequencing data to Puhti. TrimGalore (v0.6.4; https://github.com/FelixKrueger/TrimGalore; 10.5.2021) was used for the initial preprocessing. This consisted of removing low-quality bases (Phred score < 20) and Illumina Universal Adapters. Further preprocessing was performed with PRINSEQ (lite v0.20.4) (Schmieder and Edwards 2011) after which the following criteria held: mean quality score ≥ 25, read length ≥ 50 nucleotides, proportion of ambiguous bases was ≤ 1%, removal of duplicate reads, and DUST score ≤ 7. FastQC (v0.11.8; http://www.bioinformatics.babraham.ac.uk/projects/fastqc/; 10.5.2021) was used to obtain the quality scores. Alignment of the preprocessed reads against human reference genome (GCF_000001405.26_GRCh38_genomic.fna from NCBI) was performed with STAR (v2.7.1a) (Dobin et al. 2013). Finally, HTseq (Anders et al. 2015) was used to obtain the aligned HERV-W read counts. Normalization of the HERV-W read counts was performed with DESeq2 default normalization (Love et al. 2014). Read count quantity ≥ 16 was considered as reliable HERV-W expression. Differential HERV-W expression was obtained with DESeq2.

### Correlation analysis

Spearman’s rank-order correlation was used to measure the association between HERV-W RNA expression and cell type proportions. P-value less than 0.05 was considered statistically significant.

### GSEA

Gene Set Enrichment Analysis (GSEA) was used to assess the potential biological functions of different HERV-W loci. The gene list was produced by calculating the Pearson correlation efficient between HERV-W RNA expression and the gene expression. This was performed for all differentially expressed HERV-W loci. The GSEA function used was the clusterProfiler software package version 3.12.0 (Yu et al. 2012). The genome annotation was retrieved with R package org.Hs.eg.db (Carlson 2017). The minimum and the maximum sizes of gene sets were 25 and 500, respectively. Permutation size was set to 100,000 and the significance of enrichment was determined by a Benjamini–Hochberg adjusted p-value of less than 0.05.

## Results

**Fig. 1:**
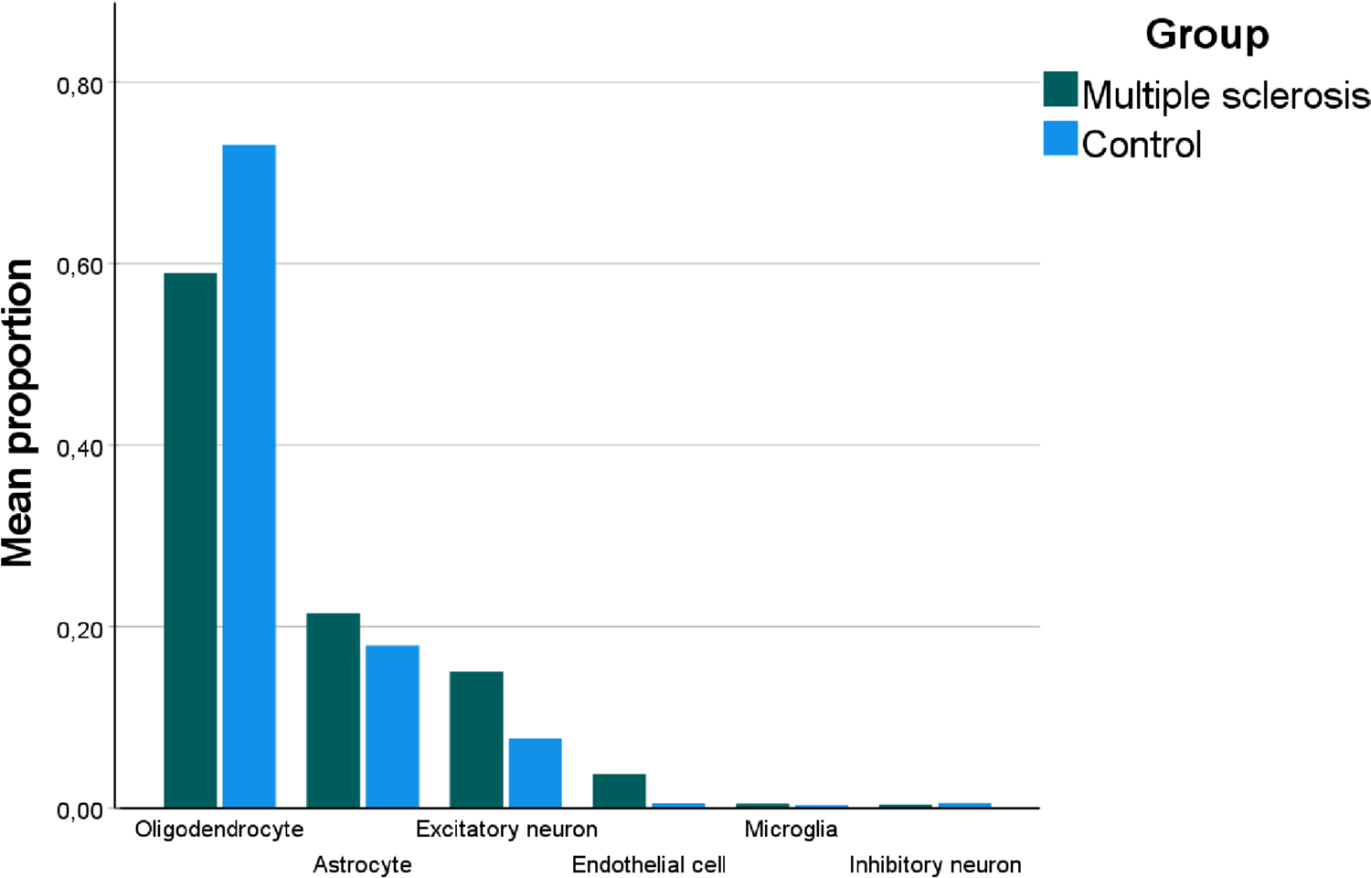
Estimation of cell type proportions of white matter brain samples. Sample population consisted of 52 MS samples and 20 control samples. Cell proportions of glial cells (oligodendrocyte, astrocyte, microglia), neuronal cells (excitatory and inhibitory neurons), and endothelial cells were estimated using CIBERSORTx algorithm. For single sample, all cell proportions add up to one. Oligodendrocytes, astrocytes and excitatory neurons were found to be most abundant cell types in white matter brain samples

The proportions of glial cells, neuronal cells and endothelial cells were estimated from the RNA sequencing data obtained from white matter brain samples. For all samples average proportions for oligodendrocytes, astrocytes, excitatory neurons, endothelial cells, microglia, and inhibitory neurons were 0.621, 0.213, 0.130, 0.03, 0.004, and 0.004, respectively. Thus, oligodendrocytes, astrocytes and excitatory neurons were the most numerous cell types, making up almost 97% of all estimated cells and oligodendrocytes were distinctly the most common cell type. To assess the differences in the cell type distributions between the MS and control samples, average cell type proportions were obtained for both groups. Cell type distributions were similar in both groups as shown in Fig. 1. However, the proportions of astrocytes and endothelial cells were significantly higher (p < 0.05) in MS individuals and proportion of oligodendrocytes was significantly lower in MS individuals as shown in Table 1.

**Table 1:** Differences in cell proportions between MS and control samples. Sample population consisted of 52 MS samples and 20 control samples. Table shows average cell proportions of glial cells (oligodendrocyte, astrocyte, microglia), neuronal cells (excitatory and inhibitory neurons), and endothelial cells separately in MS and control samples. Mann–Whitney U Test was used to assess the difference in mean ranks of the cell proportions between the groups, for which the obtained p-value is shown. Proportions of oligodendrocytes, astrocytes, and endothelial cells were significantly different between MS and control groups

**Cell type**	**Mean (MS)**	**Mean (control)**	**p-value**
Oligodendrocyte	0.585	0.725	< 0.01
Astrocyte	0.227	0.173	0.022
Excitatory neuron	0.142	0.089	0.160
Endothelial cell	0.038	0.005	< 0.01
Microglia	0.005	0.003	0.340
Inhibitory neuron	0.004	0.005	0.447

Locus-specific HERV-W RNA expression was obtained by summing up the aligned reads at each genomic location specified by Grandi et al. (2016). There was a total of 215 assessed HERV-W loci of which 65 were considered to have reliable expression (average number of aligned reads > 15). HERV-W loci 15q21.3 and 7q21.2 had overall highest expression with average of 353, and 317 reads, respectively. Rest of the loci had lower expression intensities ranging between average of 15 and 160 reads. Xq22.3 had mean read count of 31. Read counts for all studied HERV-W are provided in supplementary materials.

To assess the differences of HERV-W loci expression between the MS and control samples the differential expression analysis was performed. As shown in Table 2, a total of 14 HERV-W loci were found to be differentially expressed between MS and control samples. Of these, the majority (12) of HERV-W loci were overexpressed in MS samples (2q24.3, 2q13, 1q25.2, 2q22.2, 2p16.2, Xq13.3, 3p11.1, 4q23, 15q21.3, 6q21a, 11p14.2, and 6q21c), whereas only two loci (12q13.3 and 1q22) were expressed in lesser amounts in MS samples. The highest fold change in the difference of means was observed in case of HERV-W locus 2q24.3, where the expression was four-fold higher in the MS samples (16.74 vs 67.45 reads). One differentially expressed HERV-W locus was located in the X chromosome (Xq13.3). HERV-W locus 7q21.2, that codes for syncytin-1 was not amongst the differentially expressed loci. HERV-W locus Xq22.3, one of the putative loci for MSRV, was not observed to be differentially expressed either.

**Table 2:** Differentially expressed HERV-W loci in MS. This table shows the differentially expressed HERV-W loci between MS and control groups. RNA expression intensities were derived from RNA sequencing data and differential expression was determined with DESeq2 package in R. Sample sizes were 52 for MS group was 20 for control group. Mean values in control and MS group are provided for each HERV-W locus, along with the log2 fold change and adjusted p-value. Positive fold change indicates higher expression in MS group. This analysis revealed 14 differentially expressed HERV-W loci, of which 12 were up regulated in MS group

**HERV-W locus**	**Mean (Control)**	**Mean (MS)**	**log2 fold change**	**Adjusted p-value**
2q24.3	16.74	67.45	2.01	1.23E-08
2q13	13.39	32.73	1.33	1.41E-08
1q25.2	31.97	59.69	0.88	2.97E-05
2q22.2	8.49	26.86	1.67	8.47E-05
12q13.3	28.58	17.52	-0.70	2.56E-04
2p16.2	122.49	176.39	0.53	2.79E-04
1q22	111.93	74.83	-0.58	1.04E-03
Xq13.3	21.88	35.61	0.70	1.20E-03
3p11.1	13.69	29.57	1.12	1.32E-03
4q23	8.26	15.05	0.85	2.09E-03
15q21.3	262.33	388.98	0.57	2.22E-03
6q21a	120.32	148.73	0.31	4.47E-03
11p14.2	4.85	16.80	1.75	4.65E-03
6q21c	24.22	50.31	1.04	4.74E-03

**Fig. 2:**
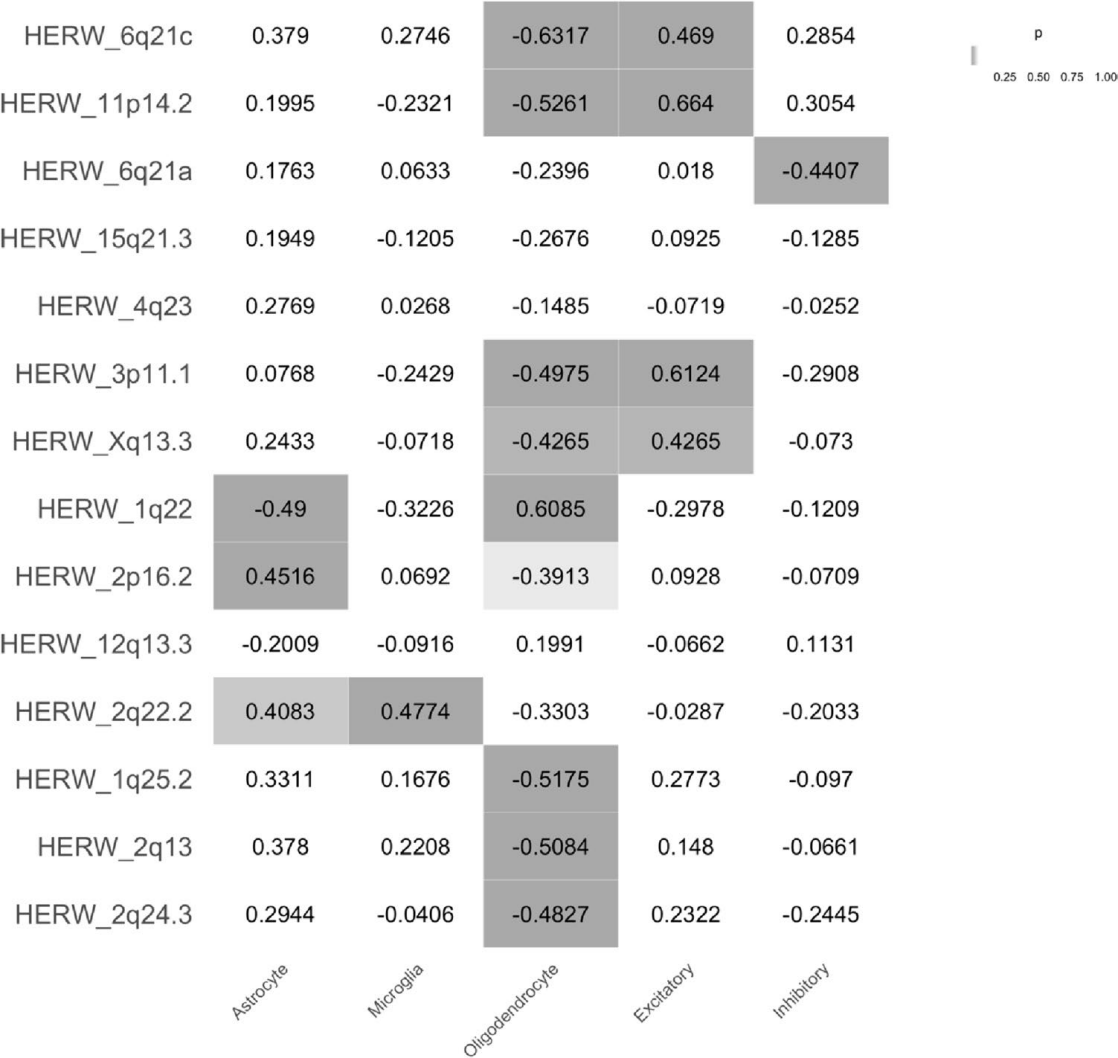
Correlation between differentially expressed HERV-W loci and cell proportions. Spearman rank correlation was utilized to determine correlation coefficients between RNA expression intensities of previously identified differentially expressed HERV-W loci (between MS and control samples) and glial (astrocyte, microglia, oligodendrocyte), endothelial, and neuronal cell (excitatory and inhibitory neurons) proportions. Background color of the tiles indicate the significance of the correlation: grey background color corresponds to significant p-values. Total of 8 HERV-W loci was identified to have significant negative correlation with oligodendrocyte proportions

The association between differentially expressed HERV-W loci RNA expression intensities and estimated cell type proportions was studied with correlation analysis. As shown in Fig. 2, significant correlations were observed in case of oligodendrocytes, astrocytes, and excitatory neurons. In case of 7 HERV-W loci (6q21c, 11p14.2, 3p11.1, Xq13.3, 1q25.2, 2q13, and 2q24.3), the HERV-W expression intensity was significantly, inversely related with the oligodendrocyte proportion. Expression intensity of only one HERV-W locus (1q22) was significantly, positively correlated to oligodendrocyte proportion. Two HERV-W loci (2p16.2 and 2q22.2) had positive, significant correlation with astrocyte proportion and one locus (1q22) had negative, significant correlation. Four loci (6q21c, 11p14.2, 3p11.1, and Xq13.3) had positive, significant correlation with excitatory neuron proportions.

**Fig. 3:**
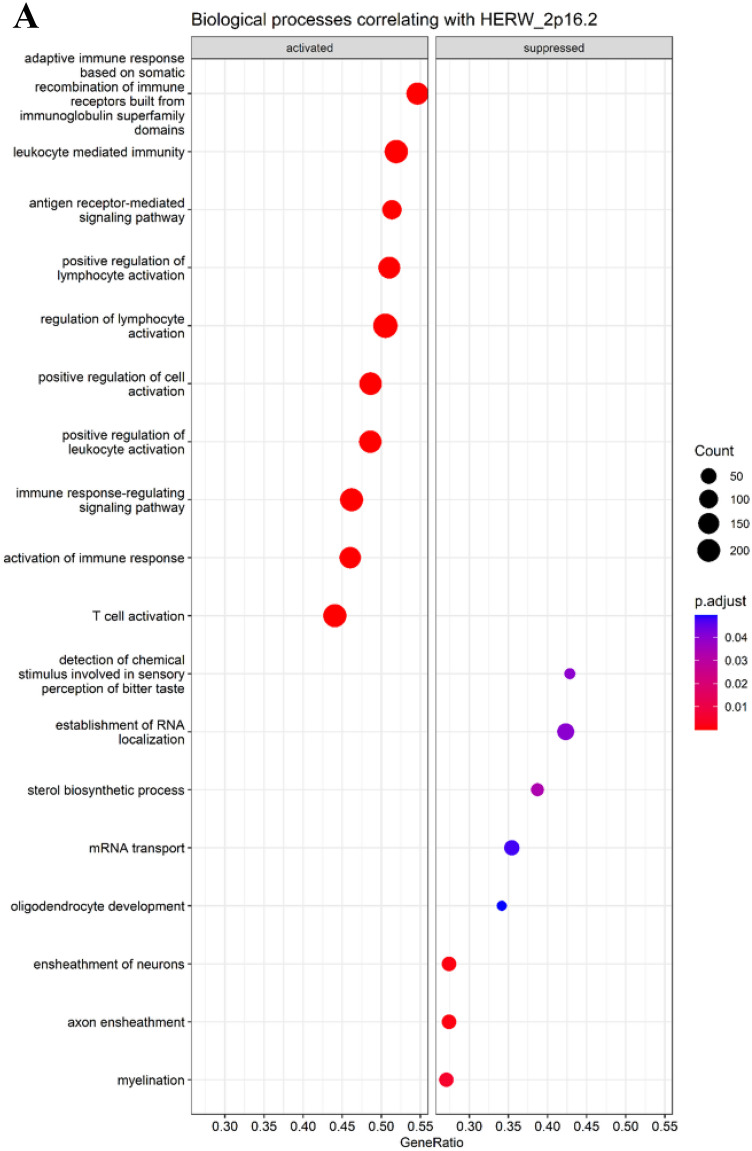

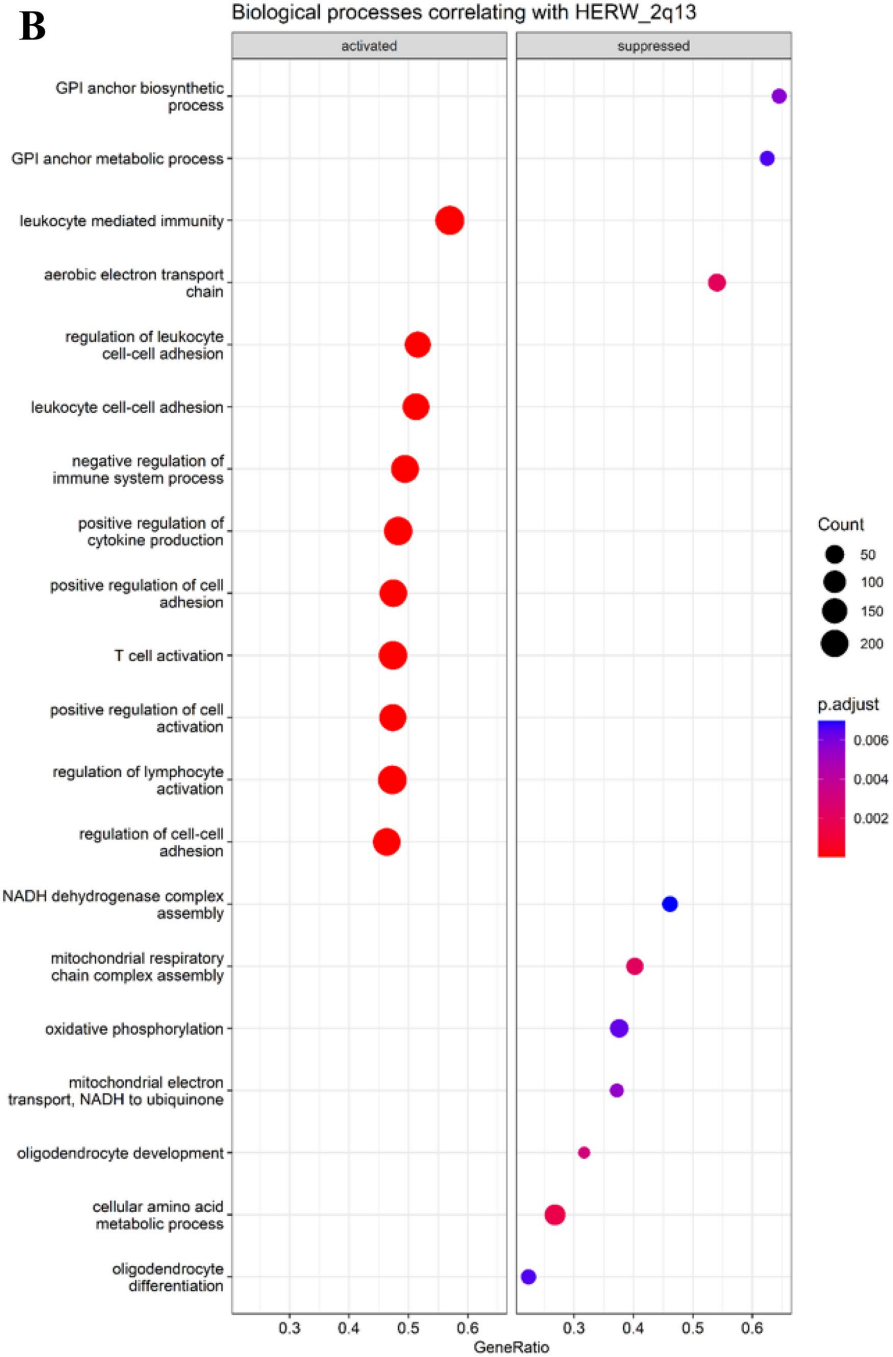

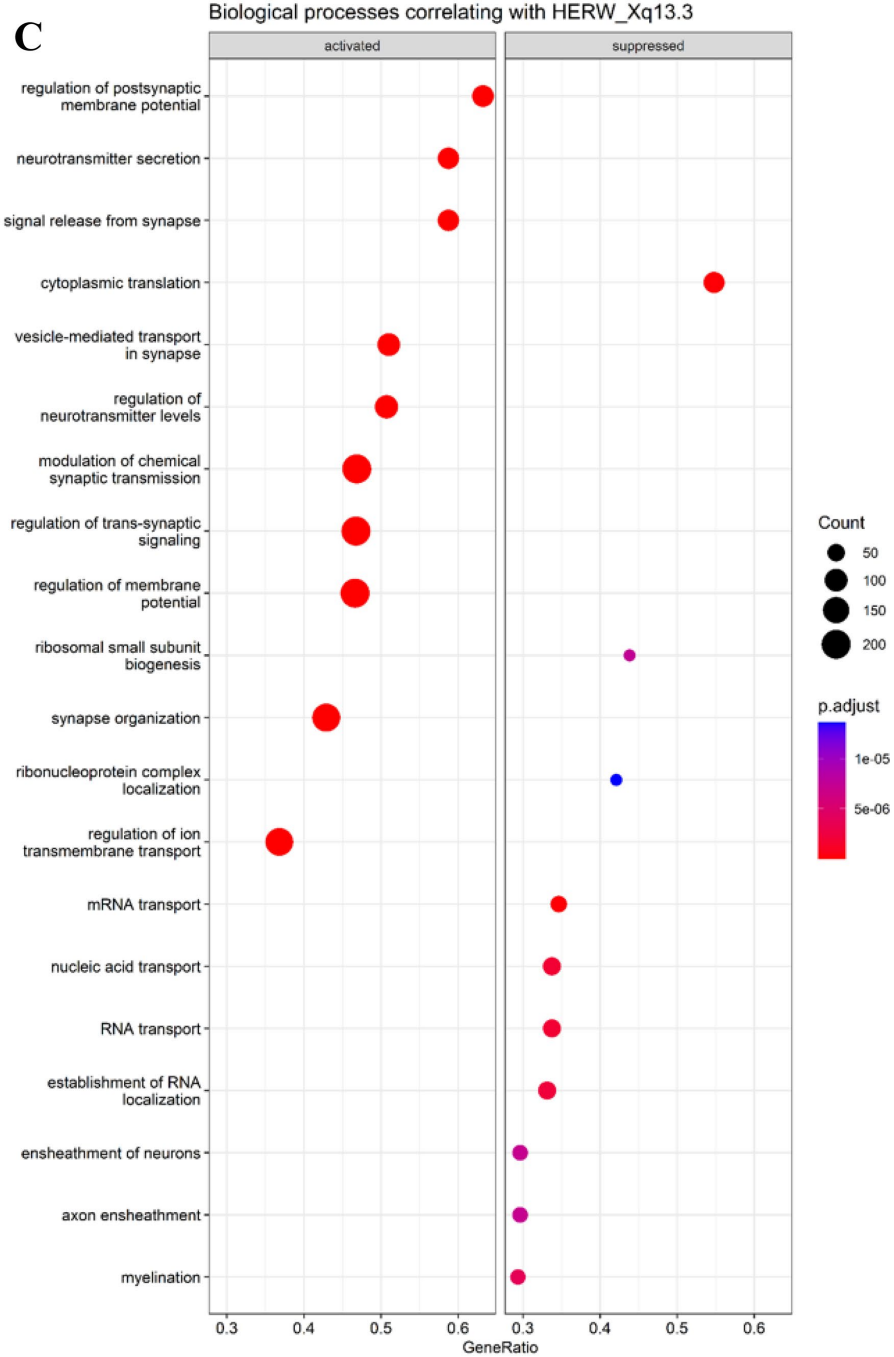
Biological pathways associated with HERV-W RNA expression intensity. GSEA was utilized to assess the biological pathways that associate with HERV-W expression. To assess this, expression levels of all differentially expressed HERV-W loci were correlated with gene expression data and this was used as an input to GSEA. Figure shows results for three selected HERV-W loci for which suppressed oligodendrocyte and myelination-related functions were identified: 2p16.2 (**A**), 2q13 (**B**), and Xq13.3 (**C**). The GSEA plots show the significant activated and suppressed biological processes. Count refers to the number of genes associated with each GO biological process. Gene ratio is the percentage of genes that significantly correlated with HERV-W RNA expression from the total number of genes associated to that process. Terms are ranked in the figure by decreasing gene ratio

Finally, GSEA was utilized to shed light into biological processes to which HERV-W expression is associated. In case of three HERV-W loci, oligodendrocyte-related functions were identified to be suppressed as shown in Fig. 3. 2p16.2 was associated with suppression of oligodendrocyte development, suppression of ensheathment of neurons, and suppression of myelination. 2q13 was associated with suppression of oligodendrocyte development and differentiation. Xq13.3 was associated with suppression of ensheathment of neurons and suppression of myelination. In addition, HERV-W loci 2p16.2 and 2q13 were associated with immune system related processes, such as T cell activation, positive regulation of cytokine production, and antigen receptor-mediated signaling pathway. GSEA results for all differentially expressed HERV-W, that had mean expression > 15 counts, are available in supplementary material.

## Discussion

Viral infections have been suggested as triggering environmental factor for MS. Especially HERV-W, a subfamily of HERVs, has been associated with MS in several studies. Although the precise pathogenic mechanisms and the specific HERV-W locus involved in MS remain unclear, it is suggested that the pathological effects are attributed to the env protein, that is translated from one or more HERV-W loci. In this study, we utilized high-throughput RNA sequencing data to investigate the transcriptional activity of 215 distinct HERV-W loci in individuals with MS and with healthy controls. Our analysis indicated that 65 HERV-W loci were transcriptionally active in the white matter of the brain. Among the expressed transcriptionally active HERV-W, 14 exhibited differential expression between MS brain samples and control samples. In addition, we found significant correlation between differentially expressed HERV-W loci and oligodendrocyte proportions in the white matter of the brain. Our findings demonstrate a substantial overlap with a previous study by Schmitt et al. (2013), where they utilized RT-PCR to quantify HERV-W locus expression and reported that 100 loci are transcriptionally active in the brain. Despite the difference in the quantification technique, the HERV-W loci that were transcriptionally most active, were remarkably similar to what we observed. In addition, both Schmitt et al. (2013) and our study identified the HERV-W locus at locus 7q21.2, which codes for syncytin-1, as having exceptionally high expression levels in the brain. These results suggest that the expression profiles of HERV-W loci in the brain are consistent across different cohorts. The differences in the number of transcriptionally active HERV-W loci can be attributed to the variation in sample size and differences in the quantification technique.

In the context of MS, neither Schmitt et al. (2013) nor our results observed upregulation of the locus at 7q21.2. The study conducted by Elkjaer et al. (2021) has also identified the HERV-W locus at 7q21.2 as the most transcriptionally active HERV-W locus and reported that it is not upregulated in MS. In contrast to these findings, earlier studies by Antony et al. (2006, 2007) have reported transcriptional upregulation of HERV-W locus at 7q21.2. Our results further challenge the initial suspicion of 7q21.2 being a source of increased env protein observed in MS. Another prime candidate for the source of env protein observed in MS is the HERV-W sequence at locus Xq22.3, which contains a nearly complete env ORF, producing truncated env protein resembling the structure of syncytin-1 (Roebke et al. 2010). Earlier studies have associated this locus as the source of env protein observed in the MS brain lesions (Rolland et al. 2006; Laufer et al. 2009; García-Montojo et al. 2014). Interestingly, our results indicated only moderate transcriptional activity (mean read count of 31) at Xq22.3 locus without significant upregulation in the MS patients. These findings suggest that Xq22.3 may not be the pathogenic HERV-W locus associated with MS. Our results are consistent with Schmitt et al. (2013), where they also did not observe MS-associated upregulation at this locus. Consequently, our study suggests, that transcriptional activities of HERV-W loci 7q21.2 and Xq22.3, previously considered prime candidates in MS pathogenicity, remain unaltered in MS.

However, we identified 12 distinct upregulated and 2 down-regulated HERV-W loci in MS patients. Three of the upregulated HERV-W loci (6q21a, 15q21.3, and 4q32.3) are considered as full-length but they have developed internal stop codons (Grandi et al. 2016). In addition, 15q21.3 was the HERV-W loci with highest transcriptional activity (mean of 353 read counts) in the white matter of brain. Interestingly, previous study by Laufer et al. (2009) has pointed out the loci 15q21.3 and 6q21c as a potential sources of env protein observed in MS. Seven of the upregulated HERV-W loci were insertions within the human coding genes: 1q25.2 (*RASAL2*), 2p16.2 (*ASB3*), 2q22.2 (*KYNU*), 2q24.3 (*COBLL1*), 6q21a (*ATG5*), 6q21c (*SLC16A10*), and 11p14.2 (*ANO3*). As for these loci, their upregulation could reflect the changes of the expression of these genes as a part of the transcriptional changes in MS. In fact, upregulation of *ATG5* has been previously reported in MS (Alirezaei et al. 2009). Three of the upregulated HERV-W loci were insertions within non-coding genes: 2q24.3 (*TCONS_00004484*), 4q23 (*LOC100507053*), and Xq13.3 (*TCONS_00016997*). In this study, we utilized RNA-seq data obtained from Elkjaer et al. (2019), who also investigated the transcriptional activity of HERV-W loci using the same dataset (Elkjaer et al. 2021). Elkjaer et al. (2021) did not observe upregulation of the HERV-W loci in MS when total reads where accounted, but found some upregulated and downregulated HERV-W loci on chromosome 7 in MS patients. In contrast, our analysis did not identify any differential HERV-W transcriptional activity in chromosome 7. This discrepancy may be attributed to the use of a different HERV-W references. Elkjaer et al. (2021) utilized more comprehensive set of references (HERVd database), whereas we used a more compact set of references by Grandi et al. (2016). It is also worth noting that Elkjaer et al. (2021) did not analyze differential expression of individual HERV-W loci outside of chromosome 7.

To assess the biological implications of transcriptional activity of HERV-W RNA in MS, we estimated the abundance of the neuronal, glial, and endothelial cells in the white matter of the brain. Plenty of studies have utilized CIBERSORTx for the deconvolution of peripheral immune cells, for which it is well validated. However, less studies have utilized CIBERSORTx for the deconvolution of central nervous system cells due to lack of well validated signature matrices. Astrocytes are often considered as the predominant glial cell in the CNS (Miller 2018). However, in the context of the white matter, the oligodendrocytes are the most abundant glial cell (Walhovd et al. 2014; Sigaard et al. 2016; Seeker et al. 2022). This is due to the role of oligodendrocytes as maintainers of myelin sheaths around neuronal axons, which reside in the white matter of the brain. Overall, the cell proportions obtained in this study are well in line with what is known about the cell abundances in the white matter. Specifically, our results highlight the predominance of oligodendrocytes in the white matter of the brain. Neurons, on the other hand, are sparsely distributed in the white matter as they primarily reside in the grey matter (Bae et al. 2020). Thus, our results further validate the performance of CIBERSORTx in estimating cell type proportions in the CNS. In the context of MS, we identified three statistically significant alterations in the cell type proportions between MS and control samples. The most prominent change was the decrease in oligodendrocyte proportions in the MS samples. This was expected as it is well known that oligodendrocyte loss is part of the MS pathophysiology (Cudrici et al. 2006). However, the specific mechanism leading to this decrease remains unclear. Both, oligodendrocyte apoptosis (Staugaitis and Trapp 2021) and the impairment of oligodendrocyte precursor cells (OPCs) (Cui et al. 2013) have been suggested to be relevant in MS. Unfortunately, we were not able to differentiate these possibilities, as the used signature matrix cannot distinguish the OPCs.

The significant increase of the astrocyte proportions in MS is consistent with findings from previous literature where proliferation and activation of astrocytes, astrogliosis, have been reported in MS (Correale and Farez 2015). Observed increase in the endothelial cell proportions could be result of peripheral immune cell infiltration to the brain due to inflammatory response. Alternatively, altered transcriptional profile might also affect how the cell type proportions are determined. While microglia also play an essential role in the MS (Luo et al. 2017), we did not observe any significant differences in microglia proportions. It is important to note that activation of microglia rather than their proportion have been reported previously in MS (O’Loughlin et al. 2018).

To assess the pathogenic potential of the 14 differentially expressed HERV-W loci, their expression intensities were correlated with the obtained cell proportions. The most prominent finding was that expression intensities of 8 HERV-W loci (6q21c, 11p14.2, 3p11.1, Xq13.3, 2p16.2, 1q25.2, 2q13, and 2q24.3) were strongly, inversely correlated to oligodendrocyte proportions. This finding suggests a potential link between the dysregulated expression of these HERV-W loci and oligodendrocyte function or survival, possibly implicating them in the pathogenesis of MS. This result was also supported by GSEA as three of the HERV-W loci were associated with suppression of oligodendrocyte-related processes, such as oligodendrocyte development and myelination. However, none of these HERV-W loci contain particularly well-preserved *env* genes suggesting that these loci do not contribute to the production of env protein observed in MS. Therefore, we found no evidence linking HERV-W loci capable of encoding env protein to the observed reduction in oligodendrocyte abundance. The study by Antony et al. (2004) presents a contrasting view showing that Syncytin-1, env protein of HERV-W at 7q21.2, is cytotoxic to oligodendrocytes. On the other hand, HERV-W mRNA has been shown to activate NF-κB and increase the production of type 1 interferons (Morris et al. 2019; Rangel et al. 2022; Russ and Iordanskiy 2023), and thus has pathogenic potential. Consequently, in theory, any HERV-W loci could be implicated in the MS pathogenesis as long as it is recognized as foreign RNA by the innate immune system. The exact mechanisms underlying the pro-inflammatory effects of HERV-W mRNA remain to be elucidated and further studies are needed to understand the molecular interactions between innate immune system and HERV-W mRNA. The underlying cause of the upregulation of the observed HERV-W is unclear. One explanation could be changes in transcriptional and/or epigenetic landscapes resulting from the disease. Alternatively, the upregulation might be attributed to a proportional increase of specific cells, that exhibit more relaxed HERV-W expression in these loci. For instance, infiltrating immune cells recruited to the CNS because of the disease might contribute to this.

There are some inherent limitations in HERV studies due to the multicopy nature and high sequence similarity between elements. One notable concern is the lack of standardized nomenclature and classification of HERVs. This can lead to inconsistencies in naming across studies and make comparisons difficult. Additionally, variety of methodologies are used for HERV detection and analysis. These include PCR techniques, RNA sequencing and bioinformatics approaches, and they can yield incomparable results. Lastly, deconvolution methods that are based on the transcriptome are still far from well-established. Namely, the lack of suitable reference profiles is a major limitation. Here we have used healthy brain transcriptome as a reference, but disease can induce gene expression changes, which could affect the cell type abundance estimates, potentially leading to inaccurate results.

In conclusion, our results indicate several HERV-W loci that are upregulated in the white matter of the brain of MS patients. Two of these loci, 15q21.3 and 6q21c, have been previously speculated to serve as potential sources of env protein. Furthermore, we found that increased transcriptional activity of eight upregulated HERV-W loci (6q21c, 11p14.2, 3p11.1, Xq13.3, 2p16.2, 1q25.2, 2q13, and 2q24.3) is associated with a decrease in oligodendrocyte proportions in the white matter of the brain. These loci do not possess well-preserved *env* genes, suggesting that the observed decrease in the oligodendrocyte abundance is not the result of the production of env protein. Thus, these findings provide new insights into the potential involvement of HERV-W mRNA in MS pathogenesis. Further investigations into the precise mechanisms are warranted to better understand their impact on oligodendrocytes and the overall disease process.

## Supplementary Information

Below is the link to the electronic supplementary material.

Supplementary file1 (CSV 8 KB)

Supplementary file2 (DOCX 2028 KB)

## Data Availability

The RNA-seq data has been made available by Elkjaer et al. (2019) (GEO, ID GSE138614).
