# Supplementary figures and images for: Human endogenous retrovirus W in multiple sclerosis: transcriptional activity is associated with decline in oligodendrocyte proportions in the white matter of the brain
Source: J Neurovirol. 2024 May 8;30(4):393–405. doi: 10.1007/s13365-024-01208-9 (PMC11512866; doi:10.1007/s13365-024-01208-9)

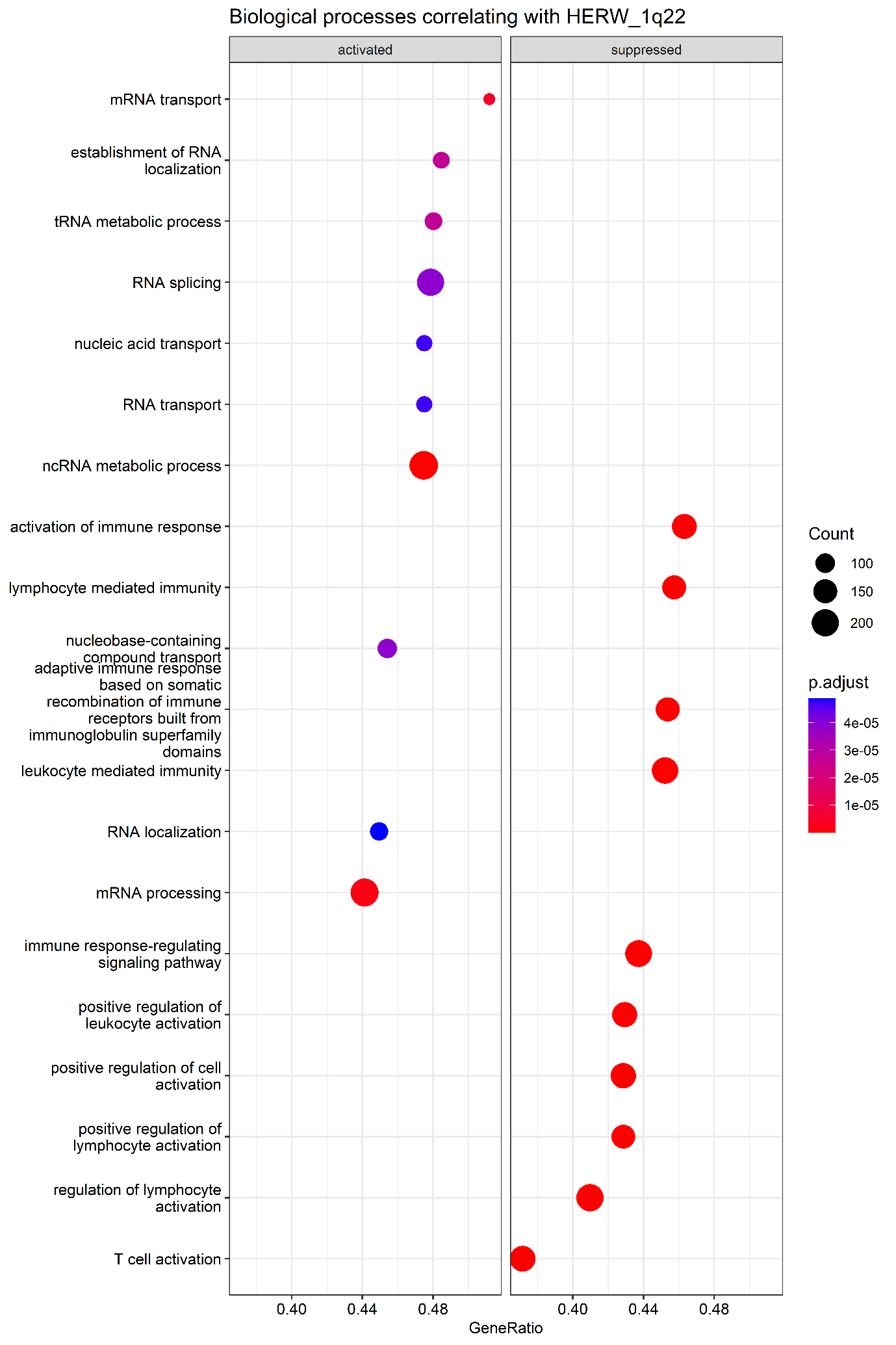

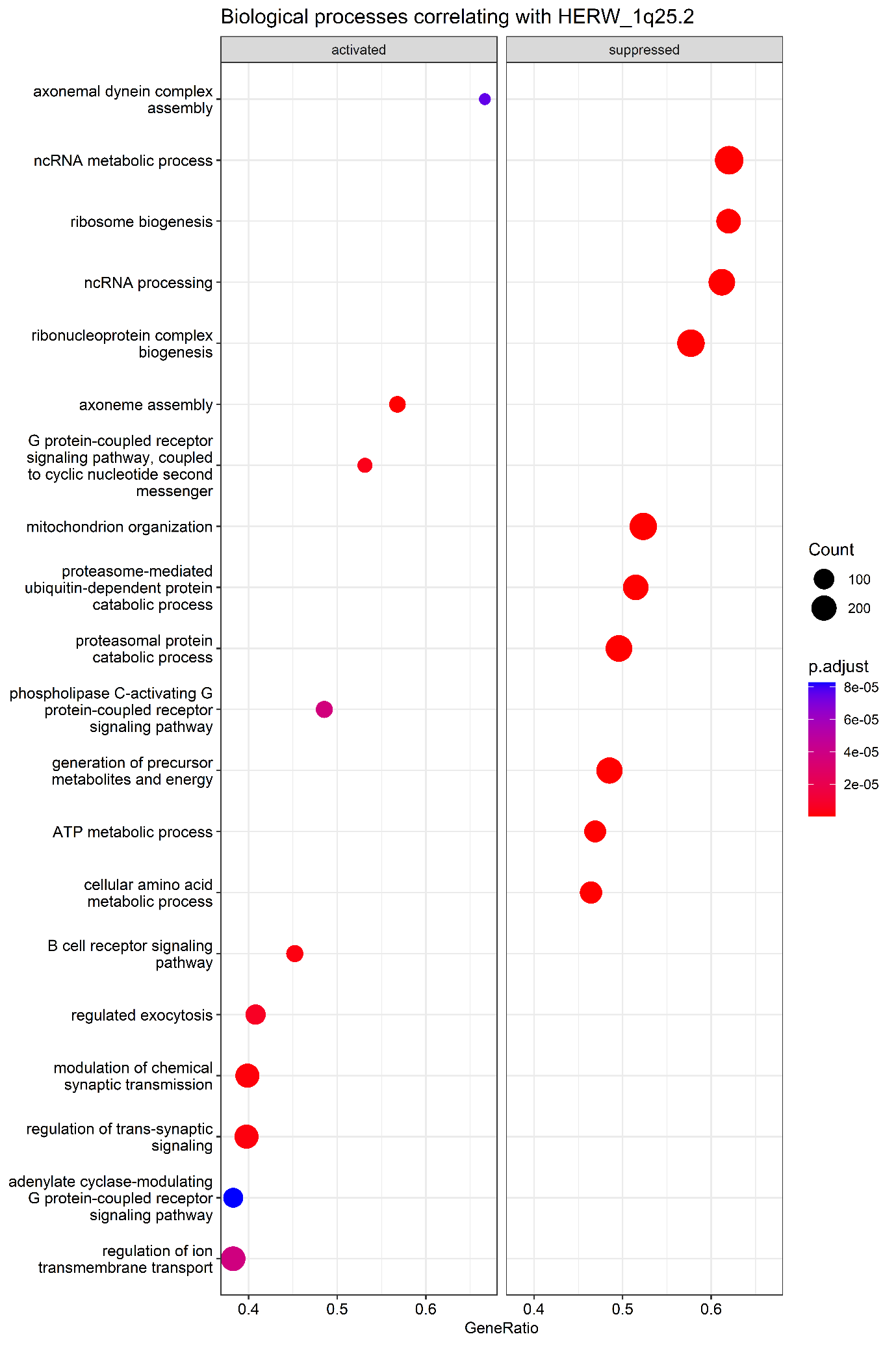

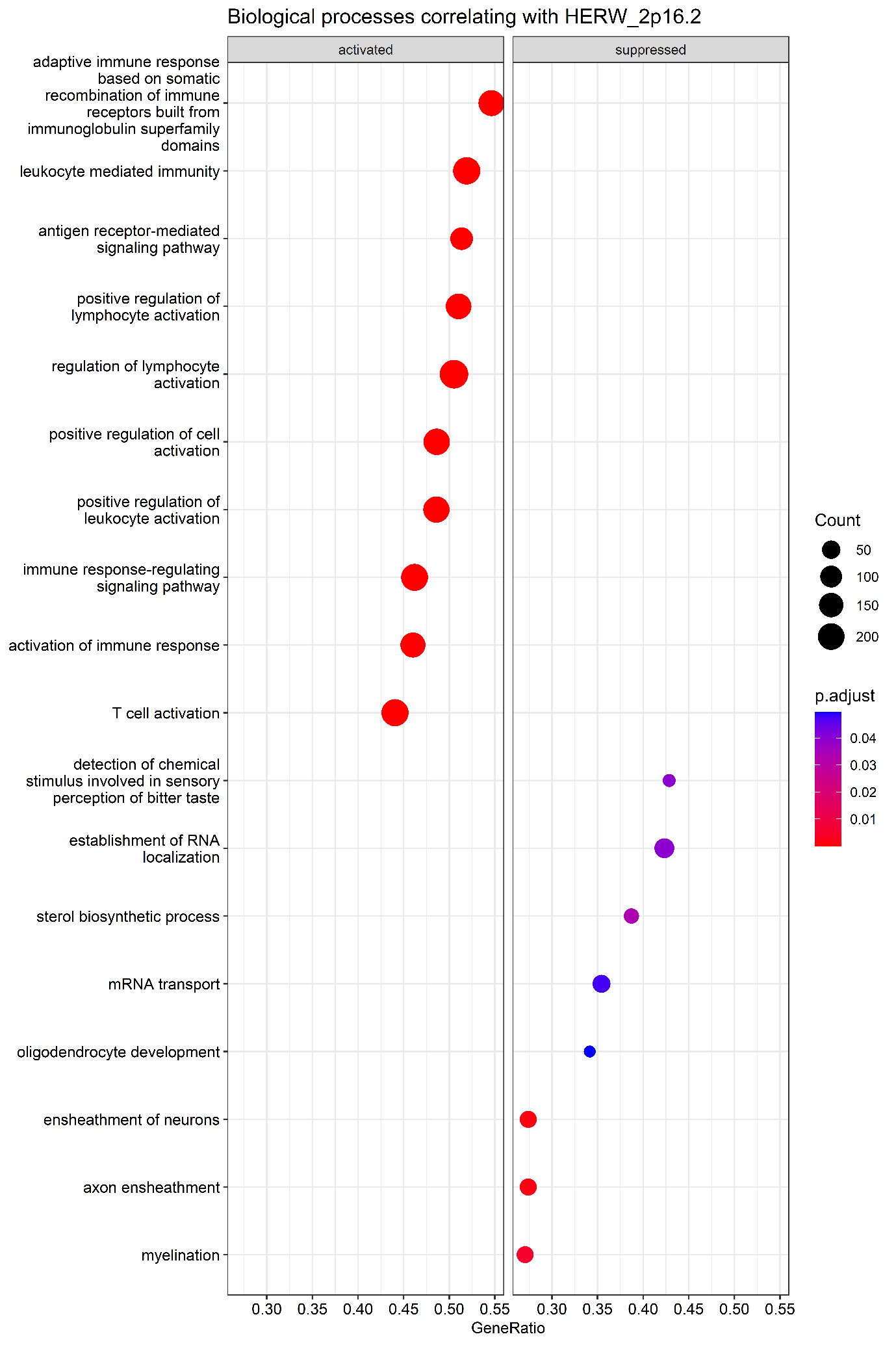

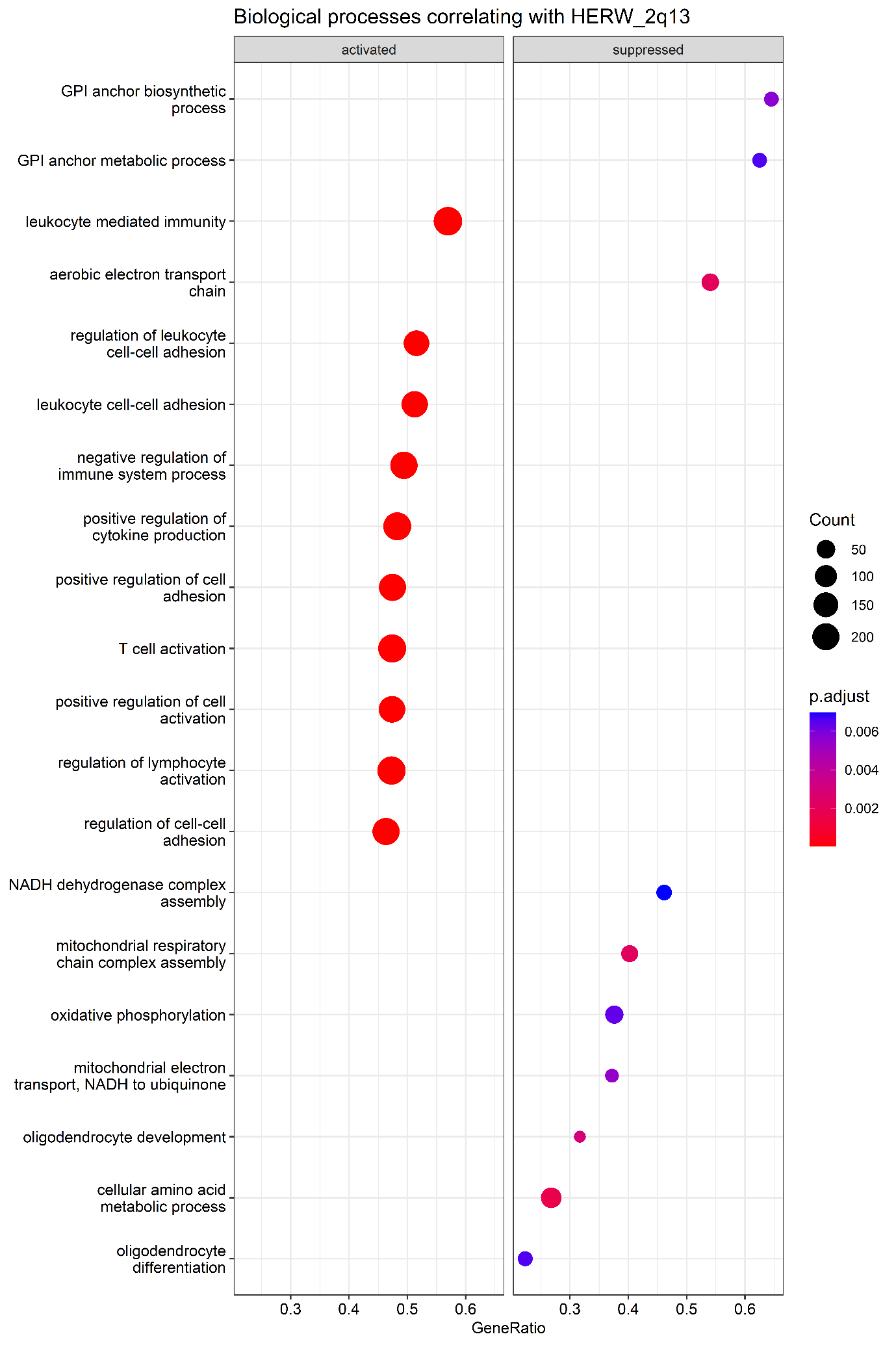

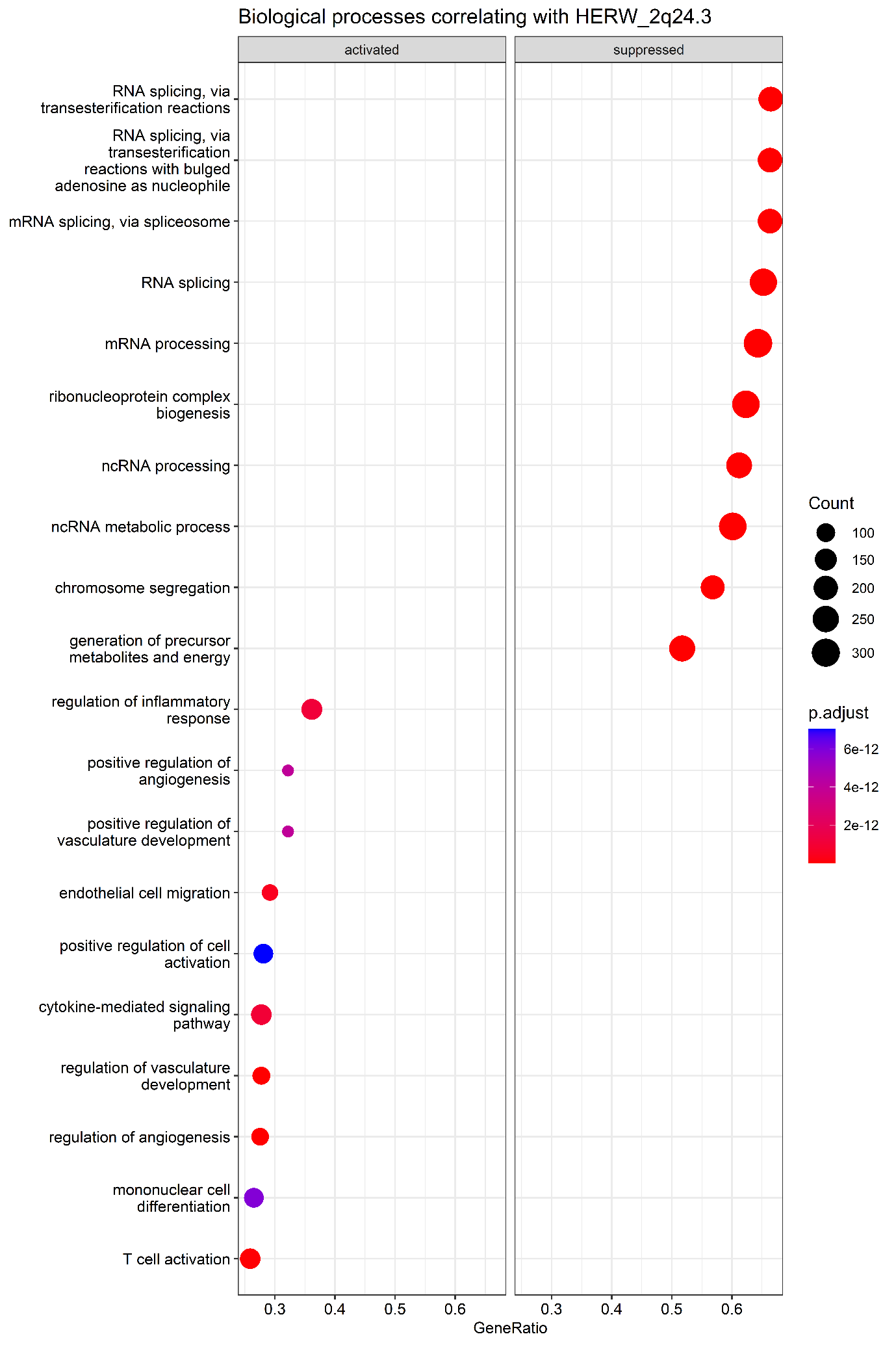

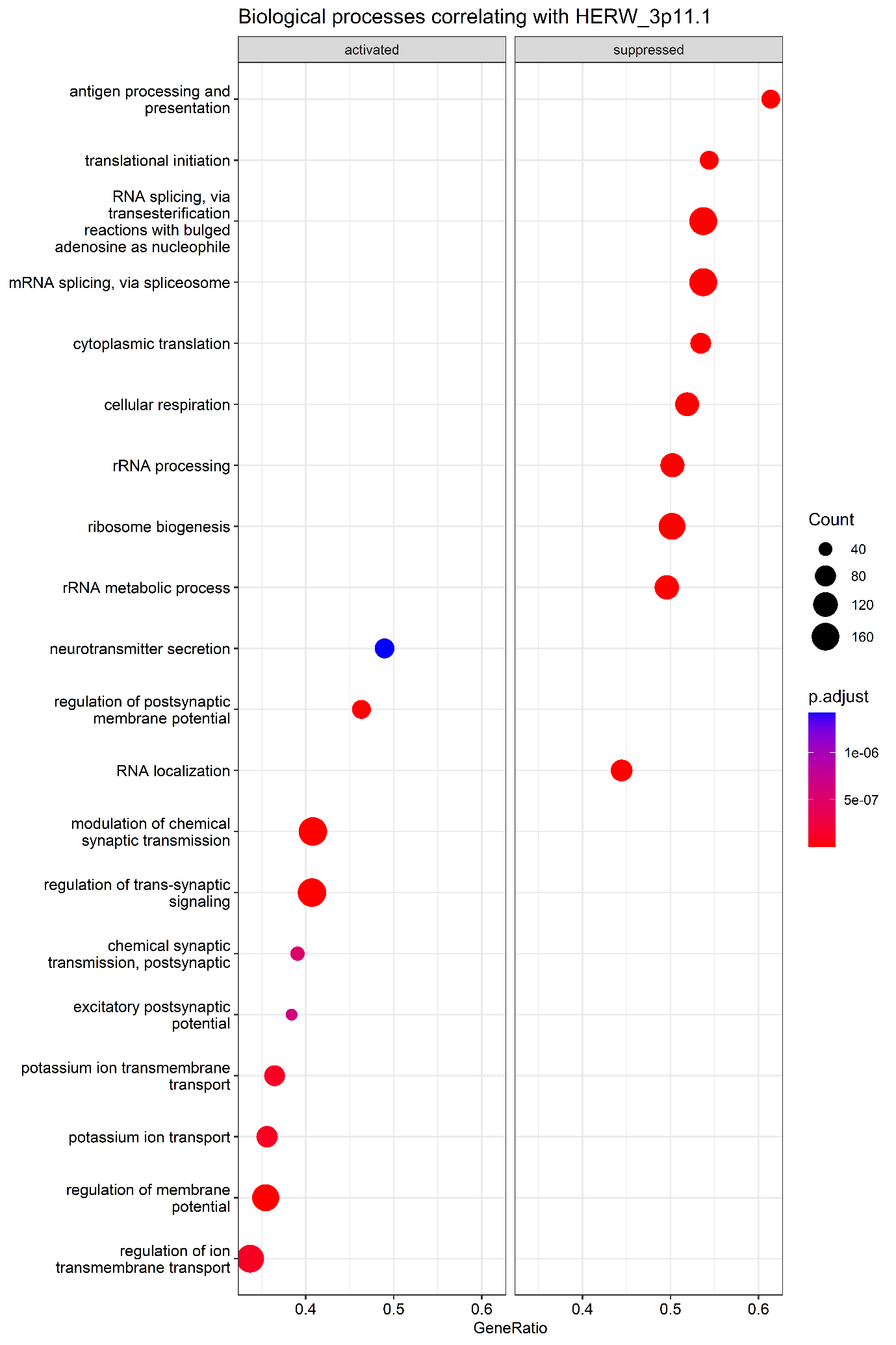

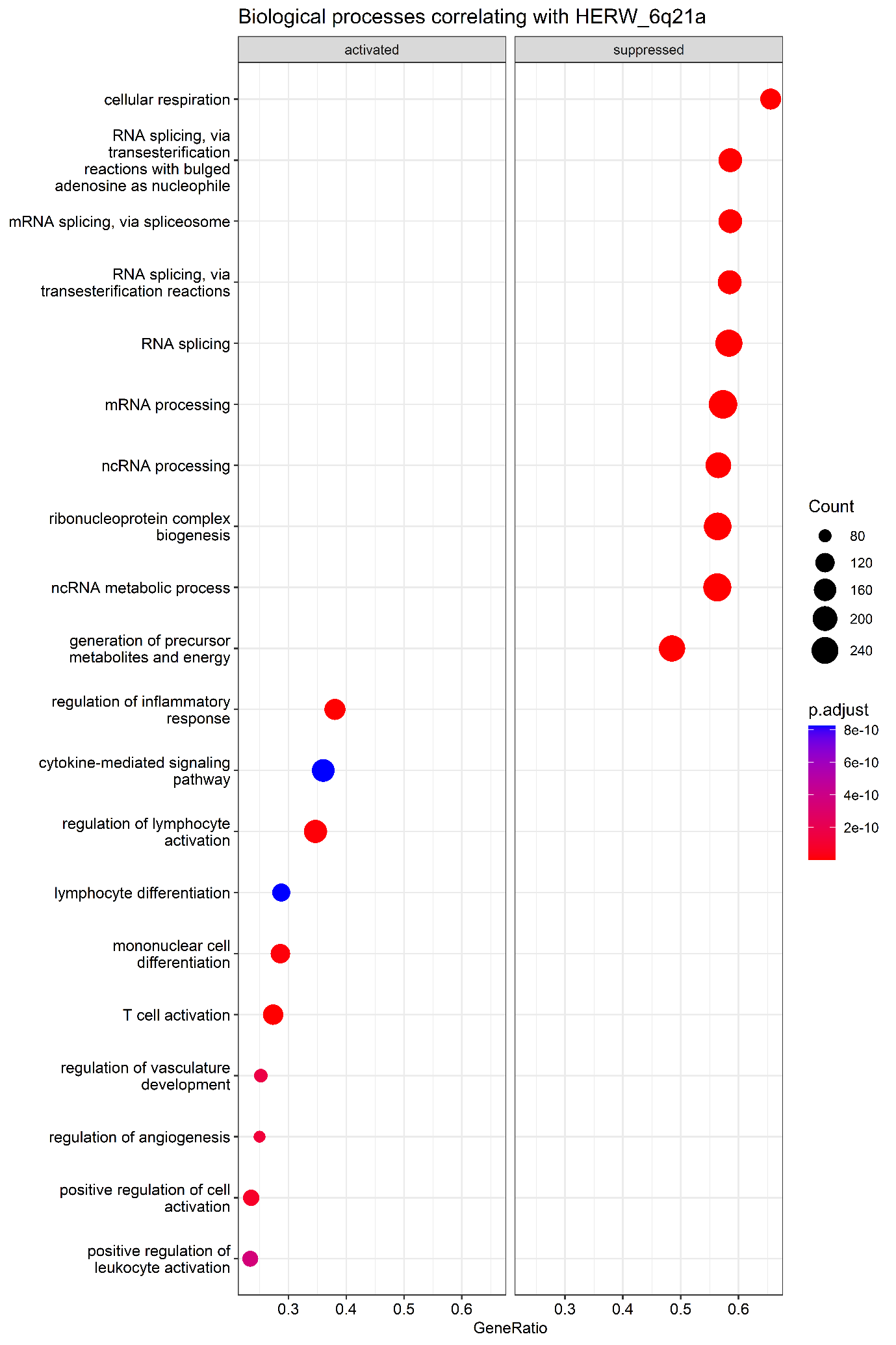

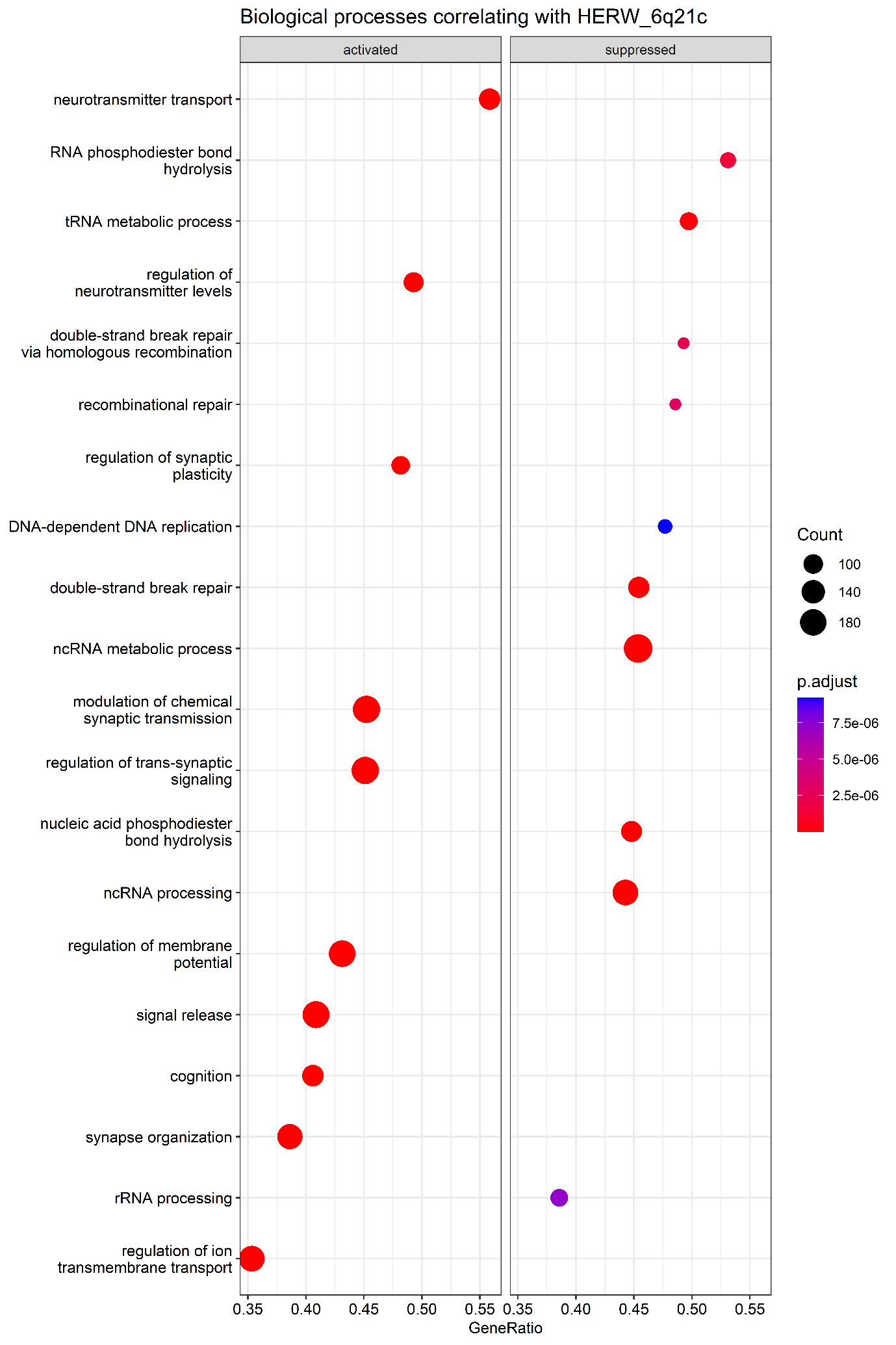

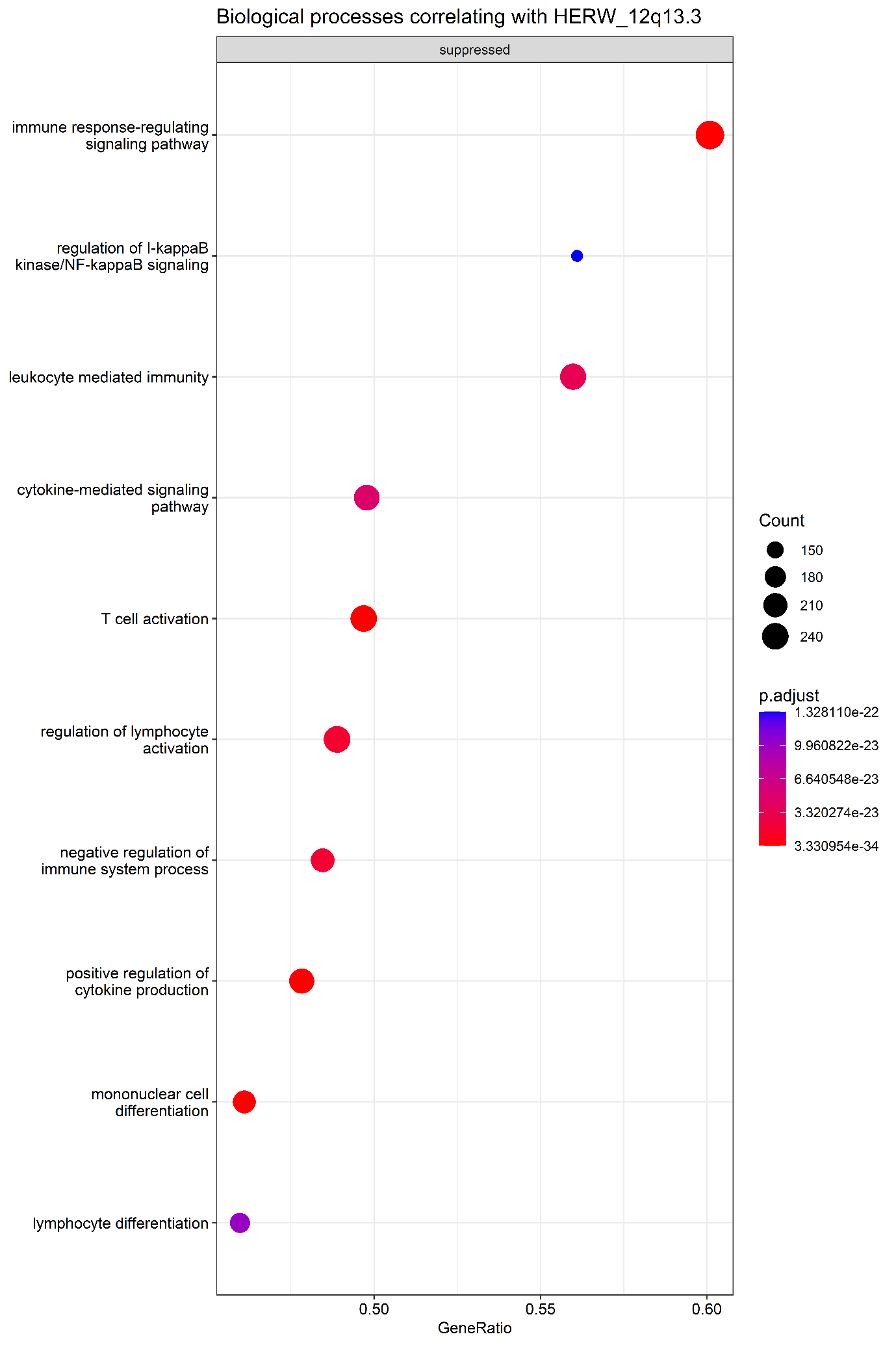

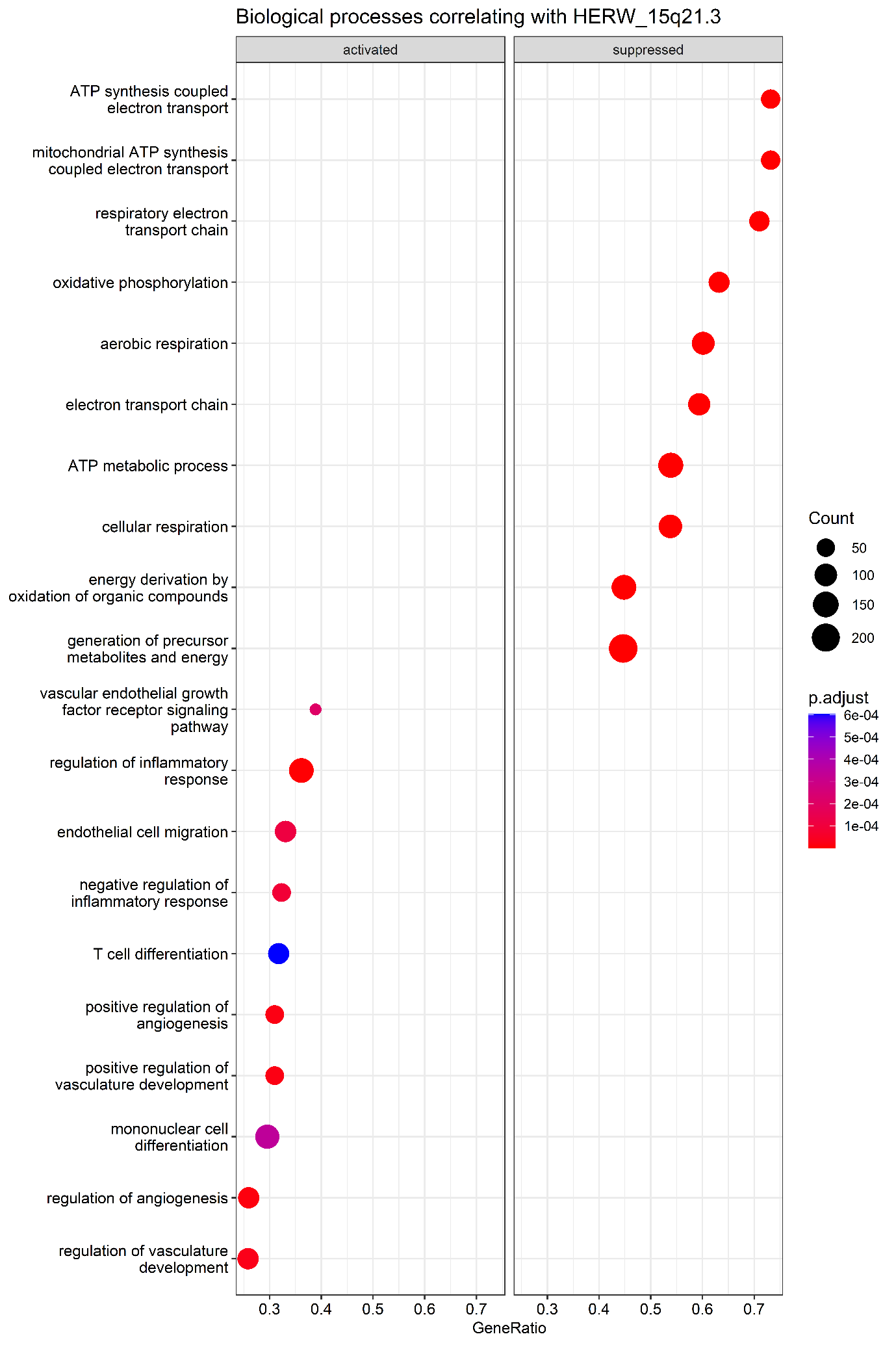

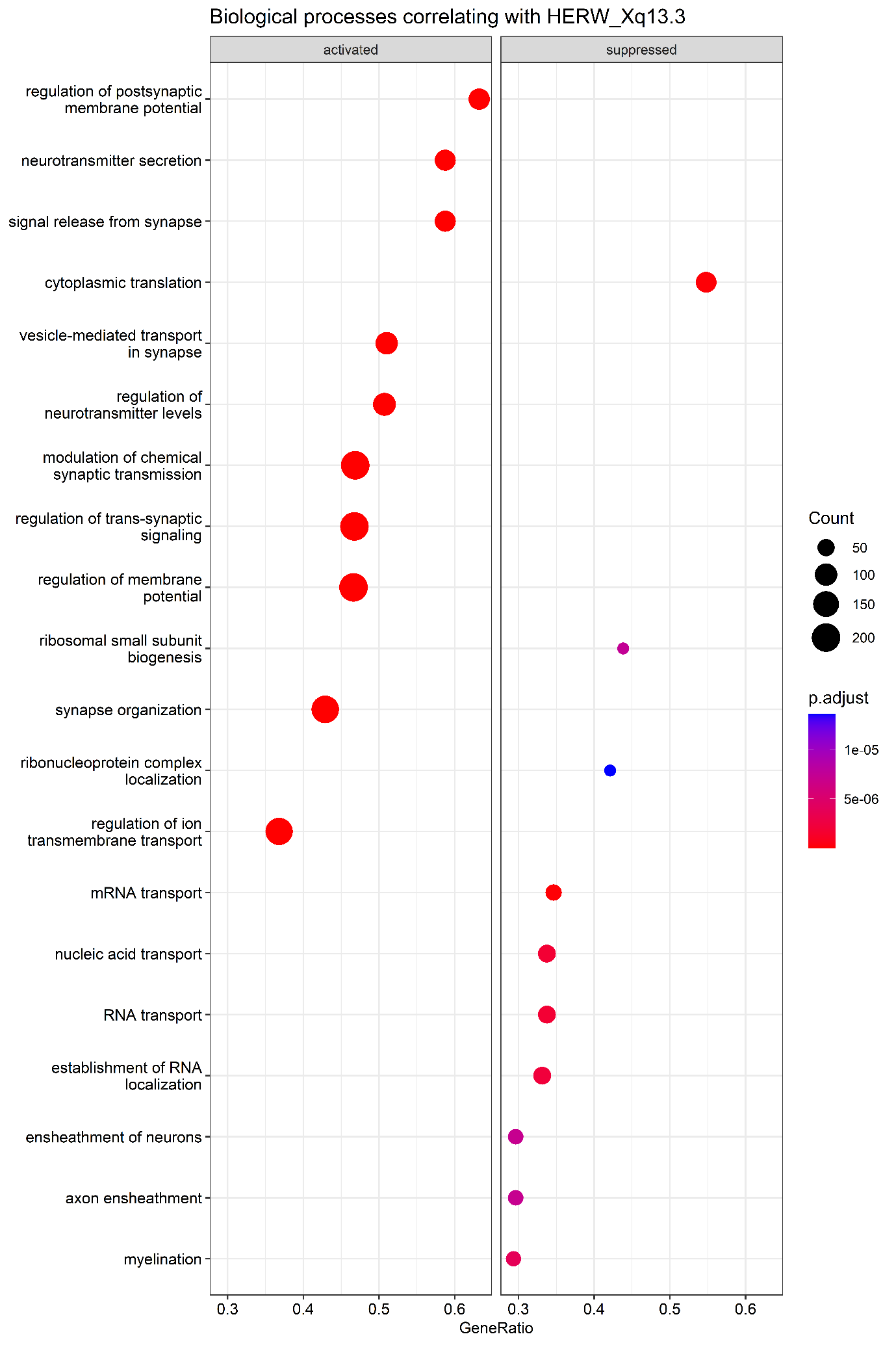

Supplement: Supplementary file 2 — Supplementary file2 (DOCX 2028 KB) [file 13365_2024_1208_MOESM2_ESM.docx]
